# Zonula Occludens Proteins Signaling in Inflammation and Tumorigenesis

**DOI:** 10.7150/ijbs.85765

**Published:** 2023-07-24

**Authors:** Sen Yu, Jie He, Keping Xie

**Affiliations:** 1 Center for Pancreatic Cancer Research, The South China University of Technology School of Medicine, Guangzhou, China.; 2 The Second Affiliated Hospital and Guangzhou First People's Hospital, South China University of Technology School of Medicine, Guangdong, China.

**Keywords:** ZO protein, tight junction, inflammation, tumorigenesis, signaling

## Abstract

Tight junction (TJ) is the barrier of epithelial and endothelial cells to maintain paracellular substrate transport and cell polarity. As one of the TJ cytoplasmic adaptor proteins adjacent to cell membrane, zonula occludens (ZO) proteins are responsible for connecting transmembrane TJ proteins and cytoplasmic cytoskeleton, providing a binding platform for transmembrane TJ proteins to maintain the barrier function. In addition to the basic structural function, ZO proteins play important roles in signal regulation such as cell proliferation and motility, the latter including cell migration, invasion and metastasis, to influence embryonic development, tissue homeostasis, damage repair, inflammation, tumorigenesis, and cancer progression. In this review, we will focus on the signal regulating function of ZO proteins in inflammation and tumorigenesis, and discuss the limitations of previous research and future challenges in ZO protein research.

## Introduction

In the course of biological evolution from single cell to multicellular organism, intercellular junctions appear and play crucial roles in various complicated and ordered biological processes. There are three categories of intercellular junctions: occluding junction, anchoring junction and gap junction. Each cell junction serves its function individually or communicate mutually to work together.

Occluding junction consists of tight junction and septate junction, two structure and function conserved junctions with some location and interactive partner variations in different species 1. Tight junction (TJ) exists in vertebrates including humans and mice and locates at cellular apical side. However, septate junction of invertebrates such as *Drosophila melanogaste*r and *Caenorhabditis elegans* locates at subapical side below adherens junction 2, 3, one category of anchoring junction, indicating that biological evolution is diverse but conservative.

TJ proteins are divided into two sorts according to their cellular location. One is transmembrane proteins comprising claundins, MARVEL domain proteins, junctional adhesion molecules (JAMs) and blood vessel epicardial substance (BVES), and the other is cytoplasmic adaptor proteins including ZO proteins, cingulin and membrane-associated guanylate kinase inverted (MAGI) proteins. Barrier function, the basic structural function of TJ, is accomplished by the cooperation of transmembrane proteins and adaptor proteins. Besides, TJ proteins participate in signal transduction to influence cell biological behaviors. Here we introduce ZO proteins, one of TJ adaptor proteins, from the respect of their structure and function, especially signal regulating roles, in physiological and pathological processes.

## ZO protein structure, basic function and functional redundancy

As cytoplasmic adaptor proteins, ZOs play a basic role in connecting transmembrane TJ proteins and cytoplasmic cytoskeleton to maintain TJ barrier function. However, functional redundancy exists among ZO proteins as a result of their structural similarity. In this part, we briefly introduce ZO protein structure and basic function, and discuss their redundant and non-redundant roles under different environments.

### Structure and basic function

ZO proteins, comprising ZO-1, ZO-2 and ZO-3, belong to TJ protein families located in cytoplasm adjacent to cell membrane. They and other adaptor proteins constitute protein plaques to provide stable scaffolds for numerous proteins including transmembrane TJ proteins, maintaining the barrier function: One is the “gate function”, which controls the paracellular transport of ions and solutes; and the other is the “fence function”, which prevents substrate mixture of the apical side and the basolateral side of one cell to keep cell polarity 4. ZO proteins are also a member of membrane-associated guanylate kinase-like (MAGUK-like) protein families, mainly containing three PDZ (postsynaptic density protein 95, discs large, ZO-1) domains, one SRC homology 3 (SH3) domain and one inactivated Guanylate kinase (GUK) domain from the amino terminus to the carboxyl terminus 1, which are crucial for the structural and the signal regulating functions of ZO proteins.

ZO proteins are indispensable for cell membrane localization and function of transmembrane TJ proteins. Although the ZO-1 knockout mouse mammary epithelial cell line EpH4 can also form integral TJ, the formation speed slows down 5. A similar phenomenon of TJ formation delay has been observed in the ZO-1 knockdown canine kidney epithelial cell line MDCK 6. Further research found that a large number of TJ proteins lost their membrane localization after simultaneously inhibiting ZO-1 and ZO-2 expressions 7, 8, which elicited enhanced paracellular permeability and therefore deregulated gate function 6, 8, 9. In the ZO-1 knockout MDCK II cell line, a monoclonal cell line of MDCK cells, subtle ZO-1 overexpression rescued deregulated TJ membrane localization, indicating that low level of ZO-1 is enough to maintain TJ formation and function 10. Although previous studies have proved that TJ is closely correlated with cell polarity 11, 12 and deficiency of all three ZO proteins triggered abnormal gate function in epithelial cells, however, cell polarity was not changed as expected 7, 8. As a consequence, which molecules are essential for cell polarity formation and maintenance remains to be further studied.

### Functional redundancy and non-redundancy

The function among ZO proteins exists redundancy due to their structural similarity. For example, in ZO-1 and ZO-2 simultaneously inhibited EpH4 cells, overexpression of either ZO-1 or ZO-2 rescued deregulated TJ membrane localization 7. In addition, *Beutel et al.* explained the functional redundancy of ZO-1 and ZO-2 from the aspect of phase separation 9. They found that most reported ZO binding proteins such as TJ proteins, cytoskeleton proteins and transcription factors enriched with ZO proteins through phase separation, and subsequent composition analysis of binding proteins suggested high similarity between ZO-1 and ZO-2, reminding their functional redundancy.

In contrast, lots of evidence proves the existence of non-redundant role of ZO proteins. *In vitro* assays reported that overexpression of ZO-2 could not rescue the TJ formation speed of the ZO-1 knockout EpH4 cells 5. Moreover, inhibition of ZO-1 rather than ZO-2 in MDCK cells promoted the paracellular permeability of larger molecules and changed cell morphology 6.* In vivo* assays provide more powerful support of the non-redundant roles of ZO proteins: First of all, the tissue distribution of ZO proteins is specific. Even if ZO-1 and ZO-2 are comprehensively distributed on the apical side of lateral membrane of epithelial and endothelial cells, ZO-3 does not exist in endothelial cells 13-15. In addition, the special structure of heart “intercalated disc” expresses only ZO-1 rather than ZO-2 or ZO-3. ZO-1 conditional knockout in mouse heart induced loss of gap junction protein connexin 40 (Cx40), leading to atrioventricular block 16. In testis, liver and inner ear ZO-2 gene mutation alone causes severe organ dysfunction 17. Moreover, either ZO-1 or ZO-2 individually knockout resulted in mouse abnormal embryonic development and embryonic lethality 18, 19. Though the embryonic development and tissue morphology were not affected in ZO-3 knockout mouse, however, ZO-3 knockout zebrafish induced improved embryonic sensitivity to osmotic pressure and epithelial permeability, contributing to abnormal embryonic development and embryonic lethality 19-21.

It should be noted that the research conclusions above are all under physiological conditions without external stimulus. Recent research has shown that ZO proteins exert extra non-redundant roles under stress, which cannot be detected in ordinary environment. For instance, the arrangement of cytoskeleton actin and microvilli of ZO-1 knockout mouse intestine only changed slightly 22. However, under chemical or immune injuries, the mucosa of ZO-1 knockout intestine was disrupted severely. Both *in vitro* and* in vivo* assays demonstrated that loss of ZO-1 restrained intestinal cell proliferation and promoted cell apoptosis. Mechanistically, ZO-1 bound centrosomes and induced correct spindle split direction in proliferating cells. Another study found ZO-2 knockout mouse liver appeared mild dysfunction and the barrier function of bile duct was not influenced 23. But under the stimulus of bile acid, the ZO-2 knockout liver was injured severely, which is possibly caused by the bile acid transport and detoxification inability of the liver. In addition, the research mentioned above that ZO-3 had a crucial role in zebrafish rather than mouse embryonic development can be explained by different stresses the two species face: Mammals living on land have only around 20 claudin genes, while teleost fish living in water with more paracellular barrier stress has around 60 claudin genes 24. In summary, ZO proteins have common redundant roles and distinct specific non-redundant roles.

## Signal regulating function of ZO proteins

In addition to the basic function of ZO proteins, they also bind various signal regulating proteins including transcription factors and protein kinases to participate in cellular signal transduction. Exploration of ZO protein signal regulating function will complement their roles in physiological and pathological processes.

### Phenomenon of nucleus-membrane shuttling

Parts of MAGUK-like proteins including all three ZO proteins have several both nuclear localization signals and nuclear export signals, hence theoretically they have the ability of moving into and out of nucleus 25. The cellular localization alteration of ZO proteins probably affects the localization of their binding partners and subsequently influences cell signal transduction. To study the nucleus-membrane shuttling of ZO proteins facilitates us to understand the mechanisms of their signal regulating function (Table 1). ZO-1 is the first ZO protein to be reported owning the ability of nucleus-membrane shuttling 26. *Gottardi et al.* found that cell density or confluency was crucial for ZO-1 cellular localization in epithelial cells. ZO-1 located in the nucleus of subconfluent cells, while it was transported out of nucleus to membrane with the rise of cell density. Afterwards, *Islas et al.* found similar cellular localization behavior of ZO-2 25. One difference between ZO-1 and ZO-2 was that ZO-2 nuclear localization could also be observed in some confluent cells, illustrating possible functional difference between them. Nevertheless, to our knowledge, any imaging evidence of ZO-3 nucleus-membrane shuttling has not been observed, even ZO-3 was detected in nuclear proteins 27. Moreover, *Balda et al.* indicated that they could not repeat the ZO-1 nuclear localization phenomenon though applying the same cell line and protein detection methods of previous studies 28. Actually, some studies have demonstrated that in confluent cells, the nuclear ZO protein levels are far lower than those of total ZO proteins 25, 26, which may cause the false negativity of ZO protein nuclear signal because it may be covered by membrane signal. As a result, more advanced technological approaches are necessary for the studying of ZO protein nucleus-membrane shuttling.

### Cell proliferation regulation

ZO proteins influence cell proliferation through nucleus-membrane shuttling and regulating cell cycle related proteins such as cyclin D1 and proliferating cell nuclear antigen (PCNA). ZO-1 mainly inhibited cell proliferation by binding transcription factor ZO-1-associated nucleic acid-binding protein (ZONAB) with its SH3 domain 28. ZONAB promoted the transcriptions of cell cycle related proteins cyclin D1 and PCNA by binding directly their promoters 28, 29. In addition, ZONAB accelerated cell cycle progression by combining with cyclin dependent kinase 4 (CDK4) and cyclin D1 facilitating them to get into nucleus 30. Consequently, when cell density is high, membranous ZO-1 sequester ZONAB in cytoplasm suppressing its nuclear localization, which inhibits cell proliferation. In contrast, when cell density is low, little membranous ZO-1 cannot sequester ZONAB effectively and cell cycle progresses normally. Similar to ZO-1, ZO-2 also mainly inhibits cell proliferation. González-Mariscal indicated that exogenous ZO-2 inhibited cell proliferation through collaborating with C-Myc and histone deacetylase 1 (HDAC1) in nucleus to form transcription regulatory complex, which bound the cyclin D1 promoter to restrain its transcription 31. Moreover, they found that ZO-2 regulated cell cycle from the posttranslational level. They observed the phenomenon that ZO-2 got into nucleus in the late G1 phase and got out of nucleus in the M phase, while cyclin D1 got into nucleus in the early G1 phase and got out of nucleus in the late S phase. Exogenous ZO-2 caused the G1/S phase delay by stabilizing the protein kinase glycogen synthase kinase 3 beta (GSK3β), which phosphorylated cyclin D1 on threonine-286 (Thr-286) to promote its degradation 32, 33. They also discovered that ZO-2 combined with transcription factor activator protein 1 (AP-1), which has been reported to own extensive cell signal regulating functions on cell proliferation, transformation and death, both on membrane and in nucleus 34, 35. Therefore, membranous ZO-2 may regulate cell proliferation through sequestering AP-1 in cytoplasm. There is little evidence and controversies suggesting the role of ZO-3 in cell proliferation. Surprisingly, cyclin D1 was observed to locate near cell membrane in dividing cells and bound with three ZO proteins in human colon epithelial cells 36. Inhibition of ZO-3, rather than ZO-2, prevented the cyclin D1 membrane localization leading to cell cycle arrest, and accelerated cyclin D1 degradation in cells treated with protein synthesis inhibitor cycloheximide, which reflected the ZO-3 functional specificity. However, the total cyclin D1 protein level kept constant in cells not treated with cycloheximide, indicating that ZO-3 may influence only a fraction of cyclin D1 near cell membrane in diving cells rather than the entire cyclin D1 protein. Besides, the protein levels and the cell localization of ZO-1 and ZO-2 did not change after ZO-3 inhibition, excluding the influence of ZO-1 and ZO-2 on cyclin D1. On the contrary, inhibition of ZO-3 promoted cell proliferation in MDCK cells 37, which may be caused by the different characteristics of the two cell lines and different experimental methods. In this study, only one stable ZO-3 knockdown monoclonal cell line was selected to be utilized for experiments. Whereas, different monoclonal cell lines are heterogeneous and possibly change their features in the process of long-term culture. Hence it is hard to say that the phenotypes observed from one monoclonal cell line are actually induced by ZO-3.

Except for cell density, the expressions and the cell localization of ZO proteins also change under external stimulus. For example, the nuclear ZO-2 protein increased without increase of the total ZO-2 protein in the condition of chemical stimulus or heat shock, proving ZO-2 nuclear import 38. Besides, in the cell wound scratch assay, the expressions of ZO-1, ZO-2 and ZO-3 decreased accompanied with ZO-1 and ZO-2 nuclear import, and the expressions of cyclin D1 and PCNA improved in the cells near the wound 25-27, 36. Because the ZO protein nuclear localization is always accompanied with stronger cell proliferation capacity, some researchers argue that in subconfluent cells nuclear ZO proteins induce cell proliferation, while in confluent cells membranous ZO proteins suppress cell proliferation 25, 36. However, the theory cannot explain all phenomena. For instance, in some highly differentiated cells which have no or weak proliferation capacity, such as intestinal epithelial cells of the top side of villi and renal tubular cells, large amounts of nuclear staining of ZO proteins can be seen 26, 39. Moreover, the studies mentioned above proved that nuclear ZO-2 negatively regulated the cyclin D1 expression suppressing cell proliferation 31, 32. Another study indicated that actually the nuclear ZO proteins increased with increased cell density 27, although their nuclear imaging was hard to observe. All above evidence opposes the theory that nuclear ZO proteins induce cell proliferation. In summary, it seems that all cells with ZO protein nuclear localization in different conditions, including low cell density, external stimulus or highly differentiated stage, undergo more or less survival stress. Therefore, it is supposed that the nuclear localization behavior of ZO proteins is possibly correlated with cell survival.

Notably, the studies of the cell proliferation regulation of ZO proteins above are most based on exogenous ZO proteins, and some exogenous ZO proteins contain only partial domains but not full-length proteins. There may be big differences between supraphysiological level of proteins or protein fragments and physiological level of integral proteins. A study has shown that ZONAB could only bind the ZO-1 protein fragment containing SH3 domain rather than full-length ZO-1 *in vitro,* and all three ZO proteins could not be found in the complexes that ZONAB bound 37. Nevertheless, another study indicated that expect ZO-3, both full-length ZO-1 and ZO-2 could bind ZONAB *in vitro* 9. The difference might be caused by the internal interaction of ZO proteins, which formed closed conformation to prevent the combination with other proteins 40, 41 and specific protein modifications or something else are possibly needed to expose their crucial binding domains. Furthermore, the cell proliferation regulation function of endogenous ZO proteins is not obvious as expected in physiological condition. In many epithelial cell lines, inhibition of neither ZO-1 nor ZO-2 or the combination of ZO-1 and ZO-2 with distinct inhibition approaches did not influence cell proliferation 5, 7, 42. However, in another study the combinational inhibition of ZO-1 and ZO-2 suppressed cell proliferation, though it did not work to inhibit ZO-1 or ZO-2 individually 37. The study indicated that ZONAB was not transported into nucleus after ZO-1 and ZO-2 inhibition but diffused or got lost in cytoplasm. These contradictory phenomena induced by endogenous or exogenous ZO proteins bring challenges to study the cell proliferation regulating function of ZO proteins. It should be considered that endogenous proteins of one family exist function redundancy because of their structural similarity. Besides, we should be cautious to face the conclusions from different research in consideration of the advantages and limitations of different experimental techniques.

### ZO protein function on epithelial-mesenchymal transition and cell motility regulation

Epithelial-mesenchymal transition (EMT) is essential for the progression of embryonic development. EMT is reactivated and plays crucial roles in damage repair, tissue fibrosis and cancer progression. In the course of EMT, epithelial cells lose cell junctions and cell polarity and rearrange their cytoskeleton leading to cell morphology alteration 43. Meanwhile the cells gain better motile ability through reprogrammed gene expression and signal transduction. Epithelium derived cancer cells with epithelial cell features can be transformed to cells with mesenchymal cell features, including enhanced abilities of migration, invasion and metastasis. Cancer cell lines derived from tumors of the same tissue, such as primary tumors or metastatic tumors, are usually various and heterogeneous with different motile abilities. Therefore, as compared with cell lines derived from normal tissues, cancer cell lines may be more suitable for studying cell motility and its regulating mechanisms.

#### Regulation of epithelial cells

In normal epithelial cells or cancer cells, adherens junction is essential for TJ formation, subsequently influencing cell motility. *Rajasekaran et al.* found that membrane localization of ZO-1 depended on E-cadherin-mediated adherens junction formation 44. In virus transfected MDCK cells, the E-cadherin expression was inhibited and membranous ZO-1 localization was lost, while the subsequent overexpression of E-cadherin rescued membranous ZO-1 localization. Mechanistically, catenins of adherens junction, including α-catenin, β-catenin and γ-catenin, combined with ZO-1 and facilitated its transport to membrane. Therefore, loss of adherens junction proteins caused by EMT may be one of the main reasons for abnormal membranous localization of tight junction proteins. The similar result was also observed in cancer cells: In the E-cadherin positive primary breast cancer cell line MCF-7 rather than the E-cadherin negative invasive breast cancer cell line MDA-MB-231, overexpression of insulin-like growth factor I receptor induced ZO-1 expression and increased cell aggregation, and the following ZO-1 inhibition prevented the course, demonstrating that ZO-1 is crucial for cell aggregation which is dependent on E-cadherin-mediated adherens junction formation 45.

#### Regulation of mesenchymal cells

In addition to motility of epithelial cells, ZO proteins also regulate motility of mesenchymal cells. Because E-cadherin gets lost in mesenchymal cells, signal regulating function of ZO-1 is independent of E-cadherin but depends on other junctional proteins, such as mesenchymal cells specific N-cadherin 46 or rearranged integrins 47. In melanoma cells ZO-1 bound N-cadherin to enhance not only adherence and invasive ability of cancer cells, but also adherence ability between cancer cells and fibroblasts 46. In subconfluent or scratched wounded cells of invasive lung cancer or breast cancer, ZO-1 was observed to co-localize with integrin α5β1. After ZO-1 phosphorylation by protein kinase C epsilon (PKCε), ZO-1 combining with integrin α5β1 was transported to the leading edge of cell membrane to guide cell migration, contributing to stronger cell invasive ability 47. Notably, the ZO-1 leading edge localization was just found in confluent cells, suggesting localization conservation of ZO-1 according to cell density. The similar leading edge localization of ZO-1 occurred in invasive colorectal cancer cells undergoing collective cell migration 48, a feature of invasive or metastatic cancer cells, which means that a group of mutually connected cells migrates to a direction as one unit without losing their cell junction 49. The study found that in invasive colorectal cancer cells, a colonic epithelium-enriched transmembrane protein PLP2 recruited ZO-1 to the leading edge of cell membrane and induced cytoskeleton actin remodeling to initiate collective cell migration.

#### Cell motility regulation through the β-catenin signaling pathway

The β-catenin signaling pathway plays indispensable roles in various biological processes, including EMT and cell motility 43, 50. Some research has shown that ZO proteins influence cell motility through regulating the β-catenin signaling pathway. *Reichert et al.* illustrated that overexpression of the ZO-1 protein fragment containing only PDZ domains diffused in cytoplasm but not be transported to membrane 51. Mechanistically, the ZO-1 protein fragment promoted EMT and carcinogenesis *in vivo* by activating the β-catenin signaling pathway. Because the β-catenin signaling pathway is always dysregulated in cancer cells, ZO-1 protein mutants may induce cancer initiation and progression. Similar cytoplasmic localization of ZO-1 could be also found in invasive breast cancer cells, while ZO-1 located on cell membrane in primary breast cancer cells 52. The researchers found that cytoplasmic ZO-1 improved cancer cell invasive ability through enhancing the expression of matrix metalloproteinase 14 (MMP14/MT1-MMP), which was associated with activated the β-catenin signaling pathway. They also indicated that both the ZO-1 protein fragment containing only PDZ domains and integral full-length ZO-1 protein could activate the β-catenin signaling pathway in invasive breast cancer cells, suggesting the functional differences of ZO-1 between normal epithelial cells and cancer cells. Afterwards they also found that ZO-2 had the same cytoplasmic localization in invasive lung cancer cells 53. However, ZO-2 inhibited the MMP14 expression and suppressed ability of cell invasion, suggesting the non-redundant functions of ZO-1 and ZO-2 in cancer cells. In addition to MMP14, IL-8 was also highly expressed in invasive breast cancer cells as compared with primary breast cancer cells 54. In primary breast cancer cells, overexpression of ZO-1 could enhance the transcription of IL-8 and ability of cell invasion, however, the course was independent of β-catenin signaling pathway, manifesting different signal regulating mechanisms between primary and invasive cancer cells with different ability of invasion.

**Figure 1:**
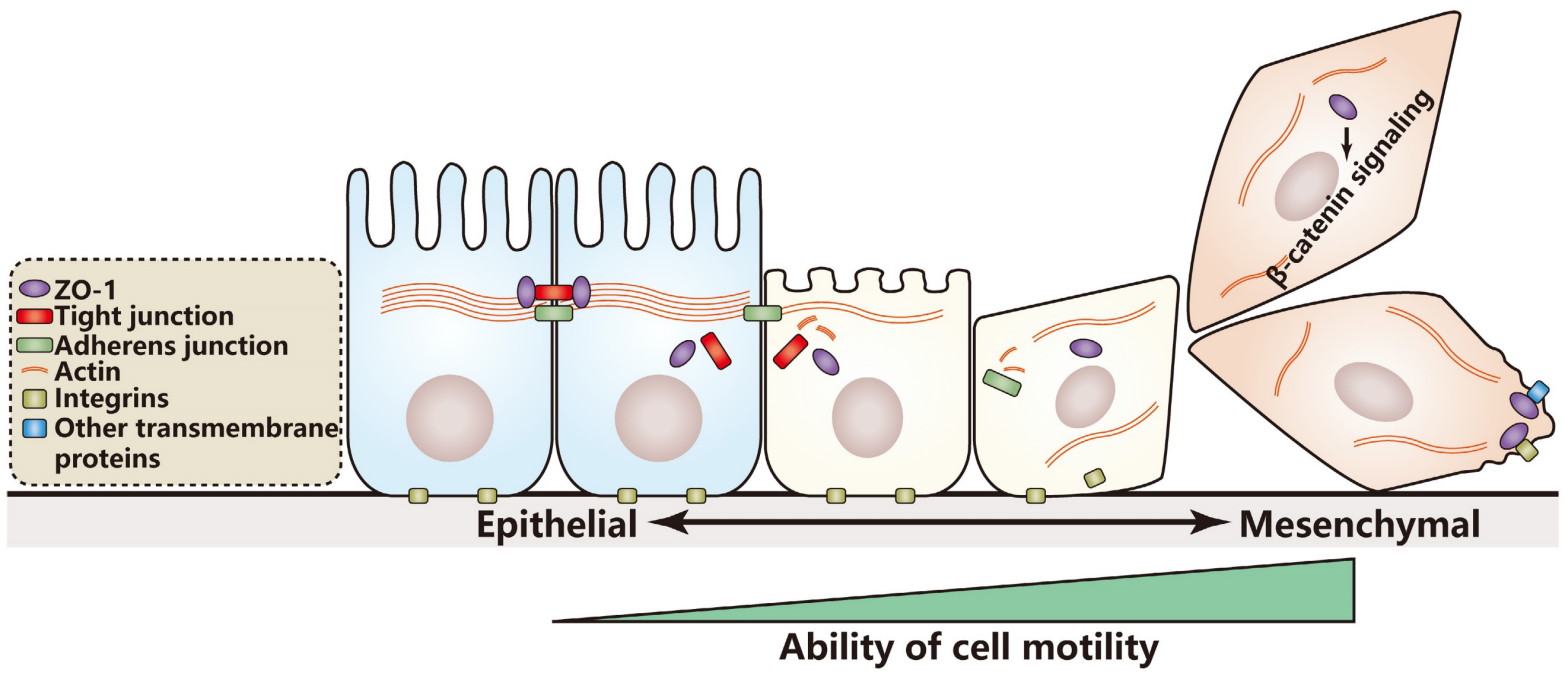
** Model for cell motility regulation by ZO-1 during EMT.** In the course of EMT, epithelial cells lose cell junctions and cell polarity and rearrange their cytoskeleton to gain better motile ability. A hypothesis is proposed that in cells with epithelial characteristics ZO-1 prefers to inhibit cell motility, while in cells with mesenchymal characteristics, ZO-1 prefers to promote cell motility through binding transmembrane proteins or activating the β-catenin signaling.

According to the studies above, a hypothesis is proposed: The influence of ZO-1 on cell motility depends on cell identity. In cells with epithelial characteristics, ZO-1 prefers to inhibit cell motility; in contrast, in cells with mesenchymal characteristics, ZO-1 prefers to promote cell motility through binding transmembrane proteins or affecting cell signaling (Figure 1). If the hypothesis is true, it will provide reference value for clinical therapy and prognosis estimation of patients with different cancer stages. However, nearly all studies on the relationship of cell motility and ZO proteins just focus on ZO-1, whether ZO-2 or ZO-3 regulates cell motility remains further research.

## ZO proteins in pathological processes

In various pathological processes such as inflammation, tumorigenesis, cancer progression and other diseases, cell identities or states change under external of internal stimuli, usually accompanied with alterations of quantity, category or binding partners of cell junction proteins. Understanding the change rules of ZO proteins in these processes possibly helps us better diagnose and treat patients.

### ZO proteins in inflammation

**Figure 2:**
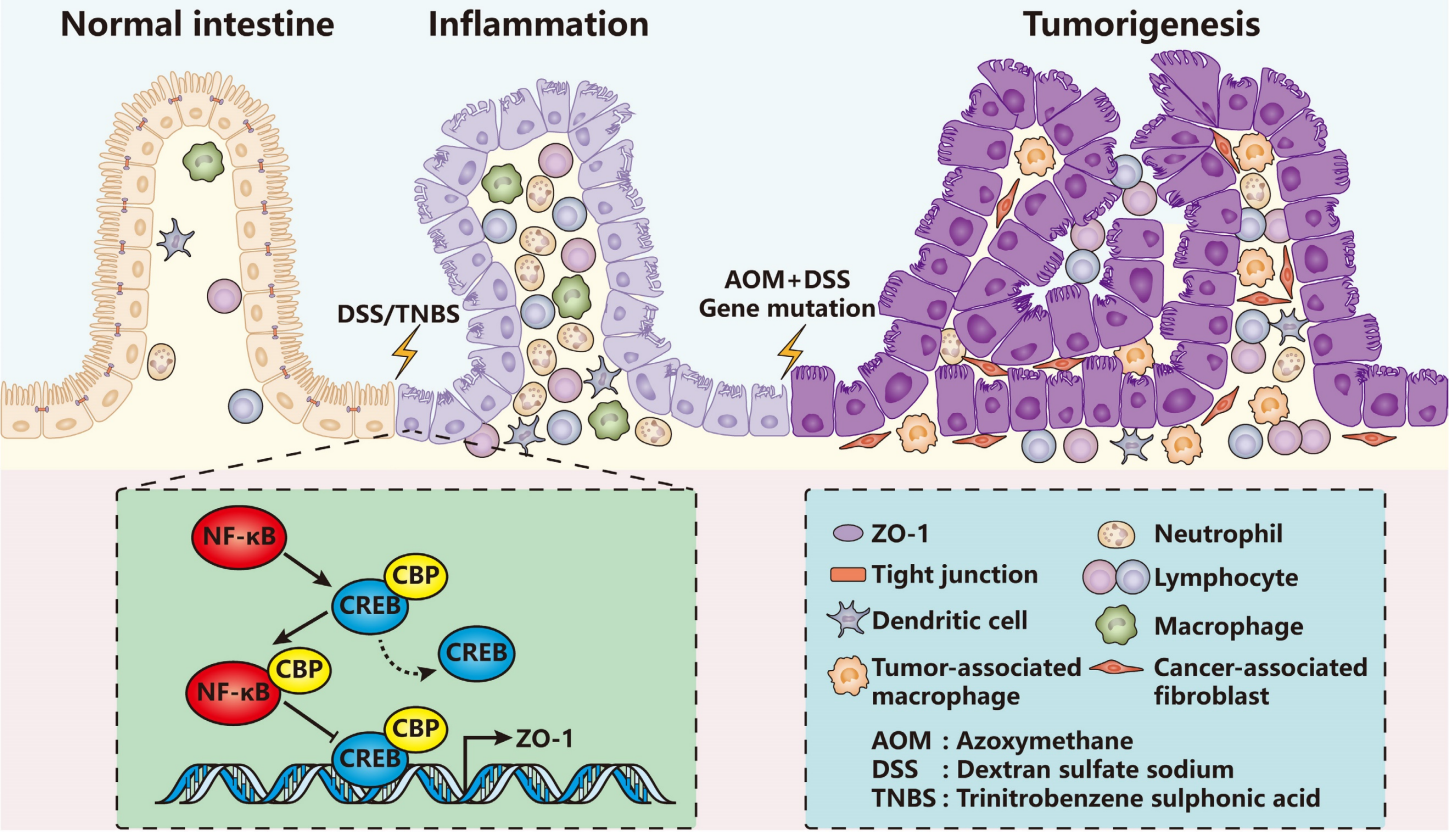
** ZO-1 signaling in intestinal inflammation and tumorigenesis.** In DSS/TNBS-induced intestinal inflammation or AOM/DSS-induced intestinal tumorigenesis, ZO-1 gets lost accompanied with severe infiltration of various immune cells. Mechanistically, inflammatory activated NF-κB replaces CREB of the CREB-CBP protein complex, which has been proved to promote ZO-1 transcription, forming the NF-κB-CBP complex to repress ZO-1 expression. In turn, loss of ZO-1 aggravates inflammatory damage and accelerates the pathological processes.

Up to now, it has been repeatedly proved that ZO proteins diffuse or get lost in nearly all types of inflammations comprising colitis 55-62, pancreatitis 63, 64, acute radiation cystitis 65, atopic asthma 66, pleurisy 67, allergic conjunctivitis 68 and so on. For example, in dextran sulfate sodium (DSS) or trinitrobenzene sulphonic acid (TNBS) induced mammary colitis models (Figure 2), the ZO-1 expression level of colon epithelia decreased quickly even on the first day 55, 57, 58. Similar results were also observed in pancreatic ductal cells in caerulein-induced mammary pancreatitis 63, 64.

ZO proteins are mainly regulated by nuclear factor kappa B (NF-κB) signaling pathway 59, 62, 69 and cytokines including tumor necrosis factor-alpha (TNF-α) 60, 67, interleukin 1 beta (IL-1β) 60, IL-6 61, IL-8 70, IL-9 68, IL-22 71 and IL-33 68. *Zhang et al.* found that in patients with inflammatory bowel disease (IBD), the expression level of monocarboxylate transporter 4 (MCT4) increased significantly 61. Overexpression of MCT4 *in vitro* disrupted intestinal barrier function via inhibiting ZO-1 expression while promoting IL-6 expression. Mechanistically, MCT4 facilitated the formation of NF-κB-CBP (cAMP-response element binding protein (CREB) binding protein) transcription factor complex, therefore preventing the binding of CREB and CBP, the combination of which has been demonstrated to play a central role in transactivation of ZO-1 72-74. Another research showed that the NF-κB activity was influenced by a transcription factor forkhead box o4 (Foxo4) *in vivo*, which reduced in the model of TNBS-induced colitis or in IBD patients 59. Foxo4 bound with NF-κB and inhibited its transcriptional activity for lots of cytokines such as TNF-α, interferon-gamma (IFN-γ), IL-1β and IL-6. Foxo4 knockout mice resulted in increased intestinal epithelial permeability and sensitivity to TNBS-induced inflammation, and down-regulation of ZO-1 and claudin-1.

Some other factors such as microbes and neurotrophic factors can also regulate the ZO protein expression. Prebiotic modulation in obese and diabetic mice under the stimulus of carbohydrate promoted intestinal epithelial secretion of glucagon like peptide-2 (GLP-2), a proglucagon-derived peptide, which protected the intestinal barrier and prevented the disruption of TJ proteins including ZO-1 and occluding 75. In addition, glial-derived neurotrophic factor (GDNF) secreted by intestinal glial cells could ameliorate DSS-induced colitis through increasing the ZO-1 expression and inhibiting cell apoptosis and cytokines such as TNF-α and IL-1β 60. Therefore, we can conclude that ZO proteins are closely associated with inflammation. ZO proteins and proinflammatory factors probably influence reciprocally.

### ZO proteins in tumorigenesis and cancer progression

It has been shown that oncogenic viruses including adenovirus and human papillomavirus can directly bind the PDZ domains of ZO proteins, therefore inducing protein degradation 76, 77. Besides, ZO proteins have highly homologous structures with another MAGUK-like protein Dlg, which has been shown acting as tumor suppressor 78, so ZO proteins are also believed to act as tumor suppressors in cancer.

Although the research on the relationship of ZO proteins and tumorigenesis is very limited, the conclusion is surprisingly consistent that ZO proteins get lost in the course of tumorigenesis 58, 79-84. The mostly used model suggesting their relationship is colitis-associated colorectal cancer, which can be induced by the combination of DSS and azoxymethane (AOM), and the expression level of intestinal epithelial ZO-1 reduces as compared with that of normal colon epithelium 58, 82-84. *Liu et al.* observed that in the mice with adenomatous polyposis coli (*Apc*) gene mutation which can develop intestinal tumors spontaneously, intestinal mucosa was damaged severely accompanied with increased tumor numbers and the loss of ZO-1 under the stimulation by deoxycholic acid 81. Furthermore, similar results were found in the course of bladder epithelial tumorigenesis of the phosphatase and tensin homolog (*Pten*) and serine/threonine kinase 11 (*Stk11*) double gene knockout mice 80. Another study showed that ZO-1 located at cell membrane in normal oral mucosa epithelial cells. However, in the cells of dysplasia or tumorigenesis, the ZO-1 expression increased and ZO-1 was transported into nucleus 85. The immune staining indicated that the proportion of ZO-1 positive cells was far more than the Ki67 positive proliferating cells, suggesting that the ZO-1 nuclear localization is not completely correlated with cell proliferation regulation again. However, the staining pattern specificity of ZO-1 remains to be testified because to our knowledge the extensive ZO-1 nuclear staining in pathological tissues has not been observed in any previous studies. The author saw the similar ZO-1 shuttling as previous studies in oral squamous carcinoma cell lines, but the difference was that ZO-1 diffused in cytoplasm rather than was transported to membrane in confluent cells 85.

ZO proteins influence cancer progression under complicated and disordered background of cancer cells. Many studies indicated loss of ZO proteins during cancer progression was always correlated with poorer prognosis 53, 86-91. However, on the contrary, in some cases high ZO protein levels accelerated cancer progression 46-48, suggesting that ZO proteins probably play different roles in different cancers or different stages of cancer progression. For instance, ZO-1 inhibition restrained oral squamous carcinoma cell proliferation and invasion 85, while improved cell proliferation and invasion capacity of endometrial cancer 92, liver cancer 93 and pancreatic cancer 94. Mechanistically, in pancreatic cancer, zinc transporter protein 4 (ZIP4) was highly expressed and inhibited ZO-1 and claudin-1 expressions by regulating a mesenchymal cell marker zinc finger E-box binding homeobox 1 (ZEB1), which bound directly to the promoters of ZO-1 and claudin-1 and repressed their transcriptions. Inhibition of ZIP4 elevated the expression levels of ZO-1 and claudin-1 and the phosphorylation levels of focal adhesion kinase (FAK) and Paxillin, two molecules related with cell adhesion and motility, while subsequent silence of ZO-1 or claudin-1 rescued the phosphorylation levels and the phenomena of attenuated cell proliferation and invasion. The controversies of cancer cell biological regulation by ZO proteins maybe explained through complicated and heterogeneous signal regulating backgrounds of different cancer cells.

### ZO proteins in other diseases

Until now, it has been ZO-2 rather than ZO-1 or ZO-3 that is closely related with other human diseases, especially for familial genetic diseases 17. For instance, familial intrahepatic cholestasis is often observed in children whose ZO-2 gene get lost due to truncating mutations 95-98. Bile of the patients does not flow through the intrahepatic ducts of the liver and some of them will develop chronic cholestatic hepatitis with cirrhosis and hepatocellular carcinoma in the late stage of the disease. Another familial disease, familial hypercholanemia, is a rare disease characterized by elevated serum bile acid concentration, pruritus, and fat malabsorption. In several Amish patients, the missense mutation V48A in the PDZ1 domain of ZO-2 reduced its stability and binding ability to claudins. In some individuals, the mutation of both ZO-2 and bile acid-CoA:amino acid N-acyltransferase (BAAT) could result in more severe structural damage and affected bile acid transport and circulation 99. In addition, the missense mutation of ZO-2 can also lead to autosomal dominant non-syndromic hearing loss 100, 101. *Xu et al.* found that the adult male ZO-2 knockout chimera mice showed reduced fertility and pathological changes in the testis whose blood-testis barrier was disrupted 99, while whether ZO-2 plays a similar role in humans remains unclear.

## Perspectives and challenges

ZO proteins play crucial roles in embryonic development, tissue homeostasis, damage repair, inflammation, tumorigenesis, cancer progression and other diseases. The structural function provides guarantee for barrier function of TJ, and the signal regulating function influences cell proliferation and motility through taking part in signal transduction. Nevertheless, what we have mastered is mainly the structural function and studies on the signal regulating function are limited. Therefore, comprehensively studying the signal regulating function of ZO proteins will help us better understand various biological processes. But before this, we have to overcome the following challenges.

The first challenge is that the functional redundancy of ZO proteins brings us difficulty to study one ZO protein individually. Previous research has reminded us that generally there are no obvious phenotypes by interfering only one ZO protein in physiological condition. In addition, the function of exogenous ZO proteins cannot represent the actual function of endogenous ZO proteins due to their differences of protein structures, expression levels and cell localization, severely obstructing our studies on the function of ZO proteins. Fortunately, the functional redundancy seems not obvious under stress, perhaps because each ZO protein participates in more complicated signal regulating processes and they cannot substitute each other. Generally speaking, more advanced research techniques and more reasonable experimental conditions are necessary.

The second challenge is that it is hard to study the function in specific place of ZO proteins because of their highly dynamic subcellular localization. From *in vitro* models we can see that not only cell density but also external stimulus can influence the localization of ZO proteins. Because the localization alteration makes it different of binding partners and protein function, it is possible to get various results in different experimental conditions. Therefore, *in vitro* model is a double-edged sword: As compared with *in vivo* model, *in vitro* model can easily reflect the localization alteration of ZO proteins in different conditions, providing more opportunities for us to study the position specific function. Whereas, the dynamic localization makes it difficult to get stable and true results. Only establishing more reasonable *in vitro* models is able to give play to the unique advantages.

The last challenge is that comprehensive distribution of ZO proteins makes them difficult to be ideal clinical drug targets. Therefore, it is imperative to figure out their functions in different tissues and their upstream and downstream regulatory modes. Otherwise, development of drug delivery approaches specific to pathological areas is requisite in the future to attenuate the side effects of drugs targeting ZO proteins. However, the amount of drug side effects targeting ZO proteins may be less than that as expected because of their functional redundancy. For example, ZO-3 is not expressed in endothelial cells and its protein function in physiological condition is very limited in mammals, it may be a more ideal therapeutic target as compared with ZO-1 and ZO-2 if ZO-3 plays crucial roles in diseases. In addition, just because of comprehensive distribution of ZO proteins and previous definite evidence of their loss under inflammation or in the course of tumorigenesis, they are expected to be ideal early clinical diagnosis markers of numerous pathogenetic processes of different organs.

## Figures and Tables

**Table 1:** Regulation of cell proliferation and motility by ZO proteins

1. Cell proliferation regulation							
ZO proteins	Protein source	Cell localization	Binding proteins	Effector proteins	Effect	Cell identity	Reference
ZO-1	Exogenous	Membrane	ZONAB	cyclin D1, PCNA	Inhibition	Epithelial	28-30
ZO-2	Exogenous	Nucleus	C-Myc	cyclin D1, PCNA	Inhibition	Epithelial	31
	Exogenous	Nucleus	GSK3β	cyclin D1	Inhibition	Epithelial	32, 33
	Exogenous	Membrane, nucleus	AP-1	-	Inhibition	Epithelial	34, 35
ZO-1 or/and ZO-2	Endogenous	Membrane	-	-	None	Epithelial	5, 7, 42
ZO-3	Endogenous	Membrane	-	cyclin D1	Promotion	Epithelial	36
2. Cell motility regulation							
ZO proteins	Protein source	Cell localization	Binding proteins	Effector proteins	Effect	Cell identity	Reference
ZO-1	Endogenous	Membrane	α, β, γ-catenins	-	Inhibition	Epithelial	44, 45
	Endogenous	Membrane	N-cadherin	-	Promotion	Mesenchymal	46
	Endogenous	Membrane	Integrin α5β1	-	Promotion	Mesenchymal	47
	Endogenous	Membrane	PLP2	RAC1	Promotion	Mesenchymal	48
	Exogenous	Cytoplasm	-	β-catenin signaling	Promotion	Epithelial	51
	Endogenous/Exogenous	Cytoplasm	-	β-catenin signaling, MMP14	Promotion	Mesenchymal	52
	Endogenous/Exogenous	Cytoplasm	-	IL-8	Promotion	Mesenchymal/ Epithelial	54
ZO-2	Endogenous	Cytoplasm	-	MMP14	Inhibition	Mesenchymal	53

## Abbreviations

TJ: tight junction
ZO: zonula occludens
JAMs: junctional adhesion molecules
MAGI: membrane-associated guanylate kinase inverted
MAGUK-like: membrane-associated guanylate kinase-like
PDZ: postsynaptic density protein 95, discs large, ZO-1
SH3: SRC homology 3
GUK: guanylate kinase
Cx40: connexin 40
PCNA: proliferating cell nuclear antigen
ZONAB: ZO-1-associated nucleic acid-binding protein
CDK4: cyclin dependent kinase 4
HDAC1: histone deacetylase 1
GSK3β: glycogen synthase kinase 3 beta
Thr-286: threonine-286
AP-1: activator protein 1
EMT: epithelial-mesenchymal transition
PKCε: protein kinase C epsilon
MMP14/MT1-MMP: matrix metalloproteinase 14
DSS: dextran sulfate sodium
TNBS: trinitrobenzene sulphonic acid
NF-κB: nuclear factor kappa B
TNF-α: tumor necrosis factor-alpha
IL: interleukin
IBD: inflammatory bowel disease
MCT4: monocarboxylate transporter 4
CREB: cAMP-response element binding protein
CBP: cAMP-response element binding protein binding protein
Foxo4: forkhead box o4
IFN-γ: interferon-gamma
GLP-2: glucagon like peptide-2
GDNF: glial-derived neurotrophic factor
AOM: azoxymethane
Apc: adenomatous polyposis coli
Pten: phosphatase and tensin homolog
Skt11: serine/threonine kinase 11
ZIP4: zinc transporter protein 4
ZEB1: zinc finger E-box binding homeobox 1
FAK: focal adhesion kinase
BAAT: bile acid-CoA:amino acid N-acyltransferase
